# STI and HIV testing: examining factors that influence uptake among domestic Australian-born, domestic overseas-born and international tertiary students studying in Australia

**DOI:** 10.1186/s12889-023-15418-z

**Published:** 2023-03-16

**Authors:** Teyl Engstrom, Michael Waller, Amy B Mullens, Joseph Debattista, Jo Durham, Zhihong Gu, Kathryn Wenham, Kirstie Daken, Armin Ariana, Charles F Gilks, Sara F E Bell, Owain D Williams, Kaeleen Dingle, Judith A Dean

**Affiliations:** 1 School of Public Health, Faculty of Medicine, The University of Queensland, Public Health Building, Herston, QLD 4006 Australia; 2 School of Psychology and Wellbeing, Centre for Health Research, Institute for Resilient Regions, University of Southern Queensland, Ipswich, Australia; 3 Metro North Public Health Unit, Metro North Hospital and Health Service, Herston, Australia; 4 School of Public Health and Social Work, Queensland University of Technology, Brisbane, Australia; 5 Ethnic Communities Council of Queensland, West End, Australia; 6 School of Health and Behavioural Sciences, University of the Sunshine Coast, Sippy Downs, Australia; 7 School of Medicine and Dentistry, Griffith University, Nathan, Australia

**Keywords:** Sexual health, STI testing, HIV testing, University students

## Abstract

**Objectives:**

Sexual health knowledge among international students in Australia is lower than domestic students, however, little is known about what factors affect the uptake of STI testing, nor if there are differences for overseas-born domestic students.

**Methods:**

We included sexually active respondents from a survey of university students in Australia (*N* = 3,075). Multivariate regression and mediation analyses investigated associations of STI and HIV testing with STI and HIV knowledge respectively, sexual risk behaviour and demographics, including comparisons among: domestic Australian-born, domestic overseas-born, and international students.

**Results:**

STI and HIV knowledge was positively associated with STI and HIV testing respectively (STI OR = 1.13, 95% CI: 1.09, 1.16; HIV OR = 1.37, 95% CI: 1.27, 1.48). STI knowledge was significantly lower for international than domestic Australian-born students (10.8 vs. 12.2 out of 16), as was STI testing (32% vs. 38%); the difference in knowledge accounted for half the difference in STI testing rates between these two groups. International students from Southern Asia, and Eastern Asia reported the lowest STI testing rates. HIV testing was highest amongst international students from Africa and North America. Higher sexual risk behaviour, younger age, and identifying as gay or bisexual were positively associated with higher STI and HIV testing rates.

**Conclusions:**

Our study supports greater investment and commitment by universities for the provision of sexual health education that can promote access to testing to improve the health of their students.

## Introduction

Young adults and overseas-born populations living in Australia are considered at an increased risk of acquiring sexually transmissible infections (STI) and blood borne viruses (BBV) including HIV. Notification rates of bacterial STI such as chlamydia, gonorrhoea and syphilis in Australia have steadily increased over the decade from 2009 to 2018 [1], with three-quarters of chlamydia infections in 2017 notified among those aged 15–29 years [1]. HIV notifications have declined over the last 5 years, however remain above average for those born overseas [1]. The majority of university students in Australia are aged below 30 [2], and almost a quarter are international students [3], suggesting the sexual health of this group should be important to university policymakers.

Migrants and international students contribute significantly to the Australian society and economy and supporting their health and wellbeing is of critical importance. Over 28% of the Australian population was born overseas; international migration increases workforce participation, brings cultural diversity, new skills and knowledge which result in new businesses, markets and fresh perspectives [4]. Permanent and temporary skilled migrants contribute $9.7 billion to the economy throughout their lives [4], and international students contributed $22 billion to the Australian economy in 2018 [3]. International students are not covered under Australia’s universal healthcare system, and are required to purchase student health insurance cover while attending university in Australia [3]. Research consistently finds international and overseas-born university students in Australia have lower sexual health knowledge than Australian-born students [5–8]. There is some evidence of lower risk sexual behaviour among international and overseas-born students from Asian countries [6, 7]. However, international students report a lack of access to health services in Australia [9], which combined with new found freedoms, stigma, costs and cultural safety create vulnerabilities to STI, HIV and other BBV [9, 10].

The asymptomatic nature of most STI means that they can be unknowingly passed onto others and if left untreated, can result in serious health outcomes such as infertility and neurological disease; hence proactive regular testing is key to prevention [11]. STI testing rates among young Australians are sub-optimal [12, 13], however, STI testing behaviour of international and overseas-born populations in Australia have not been sufficiently studied – representing potential areas of hidden public health need. To our best knowledge, there are only two small studies to date in this area: one study compared the responses of a sexual health knowledge and behaviour survey designed for Chinese International students with those from a survey targeting Australian young people and found sexually active Chinese international students had lower STI testing rates compared to domestic Australian students [7]. Similarly O’Connor et al., compared the results from different surveys in their study and found male Vietnamese migrants to Australia were less likely to have had a HIV test than other Australians [14]. Both young adults and people with culturally and linguistically diverse backgrounds (CALD) were identified as priority populations in Australia’s Fourth National STI Strategy [11]. Additionally, a HIV action roadmap for populations in Australia from high HIV prevalence countries [15] has been developed. Hence research into drivers of STI and HIV testing in domestic and international students is urgently required.

We previously published a study utilising responses from a single survey of 4,291 university students in Australia, showing that sexually active domestic Australian-born students had higher levels of STI (other than HIV, hereafter referred to as just STI) testing in the last 12 months (38.5%) compared to domestic overseas-born students (33.7%) and international students (32.4%) [8]. Domestic students are those with Australian or New Zealand citizenship, or permanent residency in Australia. However, HIV testing in the last 12 months was higher among international students (28.1%) than domestic overseas-born (23.4%) and domestic Australian-born students (22.3%) [8]. The current study expands on these findings, utilising the same survey data to explore the sexual health knowledge, sexual risk behaviour and demographic factors associated with STI and HIV testing in Australian university students. We also explored how much of the differences in STI and HIV testing rates between the three student groups are associated with sexual health knowledge, as this is a modifiable factor which studies have shown influences testing [16, 17]. This is one of the first studies to consider domestic overseas-born students and international students as distinct cohorts from their Australian-born peers, which is important as Australia’s overseas born population continues to rise and these group may have different needs and cultures related to sexual health [11]. Given the rising rates of STI in young people, persistent high HIV rates in migrant populations and the increasing contribution of international students and migrant populations to Australia’s economy and society, these findings have timely implications for the role of sexual health knowledge in STI and HIV prevention among university students.

## Methods

### Study design and data collection

A cross-sectional online survey of a convenience sample of students aged 18 and over, enrolled at five universities across South-East Queensland, was conducted between July and September 2019. This paper focuses on sexually active (ever had vaginal, anal, or oral sex) respondents.

The survey was developed by collaborating universities in partnership with local sexual and reproductive health community organisations, based on surveys and scales used in existing research involving young and CALD populations [18, 19]. The survey was administered anonymously online, an effective method to gather sensitive information from large audiences [20]. The survey took 30–40 min to complete and was in English (English proficiency is required for international students in Australia [3]), using REDCap (Research Electronic Data Capture) [21]. Engagement was incentivised through an opt-in prize draw for one of five $100 grocery e-vouchers.

Participants were recruited through university websites, social media, and campus events at all participating universities; direct email was also used at three of the participating universities, where policies allowed. Part way through data collection, the number of male respondents was low, and targeted online and peer recruitment was conducted to improve sample representativeness.

### Measures

This study considered two outcomes – STI testing in the last 12 months and HIV testing in the last 12 months. Last year testing was chosen as it more likely reflects students’ behaviour during university compared to lifetime testing. Students were categorised as domestic Australian (including students born in New Zealand) born students (DABS), domestic overseas-born students (DOBS) or international students (IS), as the primary exposure of interest, based on self-reported enrolment type and country of birth. Australian and New Zealand born students were grouped due to geographic, cultural, language and health system similarities. Demographic factors considered were: age, gender, sexual orientation, religion, living situation, relationship status, degree type, enrolment status and year of study. STI and HIV knowledge were measured with true/false/not sure questions (STI 16-items, e.g. “Human Papillomavirus can cause cervical cancer in women”; HIV 12-items, e.g. “You can get HIV from mosquitoes”). Aggregate STI knowledge and HIV knowledge scores were created, assigning one point for each correct answer [18, 22]. Internal consistency of knowledge scales was assessed using Cronbach’s α. Separate sexual risk behaviour scores (SRBS) were created for regular and casual partners as research shows IS are less likely to have casual relationships [7, 9]. Regular partners are those within a regular planned sexual relationship expected to continue; casual partners are those engaged in sex with no plan or expectation of further or long-term contact. Each SRBS score was based on four items with higher scores indicating sexual behaviours associated with increased risk of STI and HIV acquisition [23, 24]. A scale point was given for each vaginal sex partner in the last 12 months (capped at 5). An additional scale point was given for students who only sometimes used condoms and two points for those who never used condoms. The same rules were applied to anal sex encounters and added to the vaginal sex score to form the SRBS (potential range 0–14). These rules were applied consistently across all genders and sexual orientations.

### Statistical analysis

The data were analysed using Stata for Windows, Version 15. A statistical significance cut off value of *p* < 0.05 was used. A demographic summary was produced using chi-square (categorical variables) and ANOVA (continuous variables) tests for statistically significant differences between student categories. With STI and HIV testing in the last 12 months as the dependent variables, univariate regressions were built with each independent variable and significant variables were included in multivariate regressions; multiplicative interactions between sexual health knowledge and SRBS with student category were investigated. Only students who responded to the outcome variables were included in regression analyses; there were no missing responses for independent variables.

Mediation analysis was performed to understand whether being an IS impacted STI and HIV testing both directly and indirectly through STI and HIV knowledge. Interactions between student category and STI and HIV knowledge were included in the model if significant, as were significant confounders from the multivariate regression. Mediation analysis was conducted in Stata’s PARAMED package, using the counterfactual framework to split the total effect (TE) of being an IS on STI and HIV testing into natural direct effects (NDE) and natural indirect effects (NIE) through STI and HIV knowledge [25]. The proportion of the effect of being an IS mediated through STI and HIV knowledge was calculated as log(OR^NIE^)/log(OR^TE^) [26]. Mediation analysis was also conducted comparing DOBS to DABS and DOBS to IS.

## Results

### Sample statistics

From approximately 200,000 students enrolled at the five target universities [27], 4,291 responses were collected, of these 3,075 (72%) reported being sexually active ever. Sexual activity was higher in the DABS cohort (75%) compared to DOBS (69%) and IS (62%) (*p* < 0.001). Most students were aged 18–24 (67%), IS were older on average (Table 1). DABS respondents were 73% female, compared to 66% DOBS and 61% IS. IS were predominantly from Eastern Asia (27%), South-eastern Asia (19%) and Southern Asia (18%). DOBS were mainly from Northern Europe (32%), Eastern Asia (15%) and Africa (12%). STI knowledge (16 item, Cronbach’s α = 0.77) varied significantly between groups, being highest for DABS (12.2) and lowest for IS (10.8), while HIV knowledge (mean = 10.0, SD = 1.6, 12 item, Cronbach’s α = 0.63) was similar between the groups. DABS and DOBS had significantly higher SRBS with regular partners compared to IS, who reported more consistent condom use and fewer vaginal sex partners in the last 12 months. IS utilised hormonal birth control methods less (39%) than DABS (62%). DOBS were more likely than DABS to have multiple regular sex partners in the last 12 months but practiced more consistent condom use.

**Table 1:** Demographic, sexual knowledge and sexual behaviour profile of sexually active students by student category

	Domestic – Australian-Born (***n***=2257)	Domestic – Overseas-born (***n***=373)	International Student (***n***=445)	***P***-value*
**Age, years n (%)**				<0.001
18 – 19	546 (24.2)	94 (25.2)	35 (7.9)	
20 – 24	1013 (44.9)	157 (42.1)	210 (47.2)	
25 – 29	359 (15.9)	36 (9.7)	124 (27.9)	
30+	339 (15.0)	86 (23.1)	76 (17.1)	
**Gender identity n (%)**				<0.001
Male	549 (24.4)	118 (31.6)	170 (38.2)	
Female	1656 (73.6)	246 (66.0)	270 (60.7)	
Non-binary / gender diverse^a^	52 (2.0)	9 (2.4)	5 (1.1)	
**Religion n (%)**				<0.001
No religion	1589 (70.4)	223 (59.8)	214 (48.1)	
Christian	475 (21.0)	86 (23.1)	86 (19.3)	
Buddhism	14 (0.6)	11 (2.9)	30 (6.7)	
Islam	5 (0.2)	10 (2.7)	21 (4.7)	
Hinduism	6 (0.3)	12 (3.2)	46 (10.3)	
Other^a^	168 (7.4)	31 (8.3)	48 (10.8)	
**Sexual Orientation n (%)**				<0.001
Straight	1549 (68.6)	267 (71.6)	347 (78)	
Gay	118 (5.2)	15 (4.0)	33 (7.4)	
Bisexual	420 (18.6)	74 (19.8)	47 (10.6)	
Other / Not sure	170 (7.5)	17 (4.6)	18 (4.0)	
**Living Arrangement n (%)**				<0.001
Parents / Family	924 (40.9)	158 (42.4)	21 (4.7)	
Partner	579 (25.7)	94 (25.2)	89 (20.0)	
Alone	116 (5.1)	22 (5.9)	42 (9.4)	
Friends	379 (16.8)	61 (16.4)	148 (33.3)	
Campus / Share house / Other	259 (11.5)	38 (10.2)	145 (32.6)	
**Relationship Status n (%)**				0.037
Single	832 (36.9)	132 (35.4)	186 (41.8)	
Partner – live out	770 (34.1)	145 (38.9)	158 (35.5)	
Partner – live in	617 (27.3)	87 (23.3)	97 (21.8)	
Other	38 (1.7)	9 (2.4)	4 (0.9)	
**Degree Type n (%)**				<0.001
Undergraduate	1882 (83.4)	287 (76.9)	211 (47.4)	
Postgraduate	294 (13.0)	59 (15.8)	203 (45.6)	
Other	81 (3.6)	27 (7.2)	31 (7.0)	
**Year of Study n (%)**				<0.001
First year	774 (34.3)	128 (34.3)	176 (39.6)	
Second year	583 (25.8)	88 (23.6)	137 (30.8)	
Third year	437 (19.4)	75 (20.1)	93 (20.9)	
Fourth year or more	463 (20.5)	82 (22.0)	39 (8.8)	
**Region of Birth n (%)**				<0.001
Australia & New Zealand	2257 (100)	0 (0)	1 (0.2)	
Eastern Asia	0 (0)	55 (14.7)	122 (27.4)	
South-eastern Asia	0 (0)	40 (10.7)	85 (19.1)	
Southern Asia	0 (0)	31 (8.3)	80 (18.0)	
Northern Europe	0 (0)	118 (31.6)	33 (7.4)	
East, West & South Europe	0 (0)	33 (8.8)	34 (7.6)	
Northern America	0 (0)	28 (7.5)	40 (9.0)	
Africa	0 (0)	44 (11.8)	15 (3.4)	
Other	0 (0)	24 (6.4)	35 (7.9)	
**STI test in last 12 months n (%)**				0.023
No	1336 (61.5)	238 (66.3)	284 (67.6)	
Yes	836 (38.5)	121 (33.7)	136 (32.4)	
**HIV test in last 12 months n (%)**				0.038
No	1687 (77.7)	275 (76.6)	302 (71.9)	
Yes	485 (22.3)	84 (23.4)	118 (28.1)	
**STI Knowledge mean (SD)**	12.2 (2.8)	11.8 (3.1)	10.8 (3.4)	<0.001
**HIV Knowledge mean (SD)**	10.0 (1.5)	10.1 (1.6)	9.9 (1.9)	0.250
**Regular partner SRBS mean (SD)**	2.5 (2.0)	2.5 (2.1)	2.1 (2.0)	0.001
**Casual partner SRBS mean (SD)**	1.3 (2.3)	1.3 (2.3)	1.1 (2.1)	0.619
**Regular sex partners last 12 months n (%)** ^**b**^				0.182
0	314 (14.4)	49 (13.5)	73 (17.3)	
1	1,439 (65.9)	227 (62.7)	272 (64.5)	
2 or more	431 (19.7)	86 (23.8)	77 (18.2)	
**Casual sex partners last 12 months n (%)** ^**b**^				0.203
0	1,403 (64.2)	236 (65.4)	270 (64.0)	
1	297 (13.6)	46 (12.7)	73 (17.3)	
2 or more	484 (22.2)	79 (21.9)	79 (18.7)	
**Condom use – regular partner vaginal sex n (%)** ^**b**^				<0.001
Never	501 (29.9)	60 (21.6)	60 (20.5)	
Sometimes	761 (45.5)	128 (46.0)	125 (42.7)	
Always	411 (24.6)	90 (32.4)	108 (36.9)	
**Condom use – casual partner vaginal sex n (%)** ^**b**^				0.205
Never	111 (16.9)	8 (7.8)	21 (17.9)	
Sometimes	274 (41.8)	46 (45.1)	47 (40.2)	
Always	270 (41.2)	48 (47.1)	49 (41.9)	
**Hormonal contraception used last vaginal sex n (%)** ^**b,c**^				<0.001
No	561 (37.6)	88 (41.5)	141 (61.3)	
Yes	932 (62.4)	124 (58.5)	89 (38.7)	

Region of birth represents UN sub-regions, except for Africa, which combines all African sub-regions and Other, which includes Central America, South America, Caribbean, Melanesia, Polynesia, Western Asia

*p-value tests for significant difference between domestic Australian-born, domestic overseas-born and international students (chi-square test for categorical variables; one-way ANOVA for continuous variables)

^a^Includes answer of ‘prefer not to answer’

^b^Not answered by all participants

^c^Only asked to women who reported vaginal sex at last sexual encounter

### STI and HIV testing descriptive statistics

Of the 3,075 sexually active students, 2,951 (96%) students provided information about their recent STI testing, 37% of whom reported having an STI test in the last 12 months. This was highest for DABS (38%), followed by DOBS (34%) and lowest for IS (32%) (*p* = 0.023). The lower testing rate among IS was driven by students from Southern Asia (13% STI testing in the last 12 months), Eastern Asia (20%) and South-eastern Asia (26%) (Table 2). IS from Asian regions also reported lower risk sexual behaviour than other IS with both regular (mean SRBS 1.6, 2.8) and casual partners (mean SRBS 0.9, 1.2), respectively.

**Table 2:** International student STI & HIV testing rates in last 12 months

Region of Birth	n (%) (***n***=419)	STI tested in the last 12 months		HIV tested in the last 12 months	
		% tested	OR (95% CI)	% tested	OR (95% CI)
Northern America	39 (9.3)	54%	Reference	46%	Reference
Southern Asia	72 (17.1)	13%	0.12 (0.05, 0.31)	22%	0.33 (0.14, 0.77)
South-eastern Asia	81 (19.3)	26%	0.30 (0.13, 0.67)	22%	0.33 (0.15, 0.76)
Eastern Asia	118 (28.1)	20%	0.22 (0.10, 0.47)	22%	0.33 (0.15, 0.71)
Northern Europe	32 (7.6)	59%	1.25 (0.49, 3.22)	16%	0.22 (0.07, 0.68)
East, West & South Europe	31 (7.4)	52%	0.91 (0.36, 2.35)	45%	0.96 (0.37, 2.48)
Africa	13 (3.1)	54%	1.00 (0.28, 3.52)	54%	1.36 (0.39, 4.79)
Other	33 (7.9)	58%	1.16 (0.46, 2.96)	42%	0.86 (0.34, 2.19)

Region of birth represents UN sub-regions, except for Africa, which combines all African sub-regions and Other, which includes Central America, South America, Caribbean, Melanesia, Polynesia, Western Asia

North America was chosen as reference group to allow odds ratios to be calculated for Asian regions, which represent the majority of international students in Australia

Total *n*=419 excludes 26 international students who did not provide information about STI and HIV testing

*OR* Odds Ratio, *CI* Confidence Interval

Of the 3,075 sexually active students, 2,942 (96%) students provided information about their HIV testing, 23% reported testing in the past 12 months. This was highest amongst IS at 28%, followed by DOBS (23%) and DABS (22%). Among IS, the highest rate of HIV testing in the last 12 months was students from Africa (54%), North America (46%), and those from East, West, or South Europe (45%) (Table 2).

### STI and HIV testing regression analysis

Univariate regression found IS had 23% lower odds of STI testing in the last 12 months compared to DABS (OR = 0.77, 95% CI: 0.61, 0.96); this was no longer significant after adjusting for other variables. Multivariate regression found STI testing in the last 12 months was significantly positively associated with STI knowledge (OR = 1.13, 95% CI: 1.09, 1.16) and SRBS with regular (OR = 1.15, 95% CI: 1.09, 1.20) and casual partners (OR = 1.27, 95% CI: 1.22, 1.33) (Table 3). Identifying as female, gay, or bisexual, as well as living with friends were significantly associated with more STI testing in the last 12 months. There were no statistically significant interactions between student category and STI knowledge or SRBS.

**Table 3:** Multivariate regression results for outcome of STI testing & HIV testing in last 12-months

Variable	STI Testing (***n***=2951)		HIV Testing (***n***=2942)	
	OR (95% CI)	***P***-value	OR (95% CI)	***P***-value
**Student Category**				
Domestic – Australia born	Reference		Reference	
Domestic – Overseas-born	0.87 (0.67, 1.14)	0.319	1.02 (0.77, 1.36)	0.889
International student	0.91 (0.69, 1.20)	0.504	1.27 (0.96, 1.69)	0.092
**STI/HIV Knowledge Score** ^**b**^	1.13 (1.09, 1.16)	<0.001	1.37 (1.27, 1.48)	<0.001
**Regular Partner SRBS**	1.15 (1.09, 1.20)	<0.001	1.08 (1.03, 1.14)	0.002
**Casual Partner SRBS**	1.27 (1.22, 1.33)	<0.001	1.25 (1.19, 1.31)	<0.001
**Age, years**				
18 – 19	Reference		Reference	
20 – 24	1.31 (1.03, 1.67)	0.031	1.18 (0.91, 1.55)	0.218
25 – 29	1.22 (0.91, 1.65)	0.183	1.12 (0.80, 1.58)	0.510
30^b^	0.88 (0.64, 1.21)	0.433	1.05 (0.73, 1.52)	0.791
**Gender Identity**				
Male	Reference		Reference	
Female	1.59 (1.30, 1.95)	<0.001	0.97 (0.78, 1.21)	0.786
Non-binary / gender diverse^a^	1.23 (0.68, 2.21)	0.501	0.98 (0.53, 1.82)	0.958
**Sexual Orientation**				
Straight	Reference		Reference	
Gay	1.94 (1.35, 2.79)	<0.001	2.27 (1.56, 3.29)	<0.001
Bisexual	1.36 (1.1, 1.69)	0.005	1.55 (1.22, 1.96)	<0.001
Other / Not sure	1.17 (0.84, 1.64)	0.356	1.05 (0.71, 1.55)	0.805
**Living Arrangement**				
Parents / Family	Reference		Reference	
Partner	0.88 (0.61, 1.28)	0.509	1.11 (0.73, 1.69)	0.639
Alone	1.14 (0.78, 1.66)	0.497	1.88 (1.28, 2.77)	0.001
Friends	1.39 (1.09, 1.77)	0.008	1.37 (1.04, 1.79)	0.024
Campus / Share house / Other	1.09 (0.84, 1.43)	0.513	1.02 (0.75, 1.39)	0.898
**Degree Type**				
Undergraduate	Not included		Reference	
Postgraduate			1.32 (1.03, 1.70)	0.030
Other			0.99 (0.62, 1.57)	0.950

Other variables included in regression analysis but not presented above due to not being significant in multivariate regression: enrolment status (full/part-time, on/off campus), year of study, religion, relationship status

*OR* Odds Rati, *CI* Confidence Interval

^a^Includes answer of ‘prefer not to answer’

^b^STI knowledge included for STI testing outcome; HIV knowledge included for HIV testing outcome

Considering HIV testing in the last 12 months, multivariate regression found that each additional correct answer in the HIV knowledge questionnaire increased the odds of testing by 37% (OR = 1.37, 95% CI: 1.27, 1.48). Each additional one unit increase in SRBS increased the odds of testing by 8% (OR = 1.08, 95% CI: 1.03, 1.14) and 25% (OR = 1.25, 95% CI: 1.19, 1.31) for regular and casual partners respectively. Student category was significantly associated with HIV testing in univariate regression, but not in multivariate regression. Students identifying as gay (OR = 2.27, 95% CI: 1.56, 3.29) or bisexual (OR = 1.55, 95% CI: 1.22, 1.96) had significantly higher likelihood of HIV testing in the last 12 months, as were those living alone or with friends and postgraduate students (Table 3).

### STI and HIV testing mediation analysis

The mediating effect of STI knowledge on the relationship between being an IS and STI testing indicates significant indirect effects, both in univariate mediation analysis and after adjusting for confounding factors that were significant in multivariate regression (SRBS, age 20–24, female, gay, bisexual, living with friends) (Table 4). As stated above, the STI testing rate in the last 12 months is lower in IS compared to DABS (32% vs. 38%). Mediation analysis estimated that 55% of the difference in STI testing rate of IS was explained by these students having a lower level of STI knowledge than DABS. DOBS had a slightly lower average STI knowledge score than DABS (11.8 vs. 12.2), they also exhibited lower STI testing rates in the last 12 months (34% vs. 38%). Mediation analysis suggests STI knowledge accounted for 20% of the difference in STI testing rate between DOBS and DABS.

**Table 4:** Mediation effects of STI knowledge on international and domestic overseas-born students STI testing in last 12 months compared to domestic Australian-born students with confounding variables

Effect	Domestic Australian-born students (*n*=2172) compared to:			
	International students (***n***=420)		Domestic overseas-born students (***n***=359)	
	OR (95% CI)	***P***-value	OR (95% CI)	***P***-value
Natural direct effect	0.89 (0.70, 1.14)^a^	0.373	0.88 (0.65, 1.10)	0.210
Natural indirect effect	0.87 (0.83, 0.92)	<0.001	0.96 (0.92, 1.00)	0.035
Total effect	0.78 (0.61, 1.00)	0.048	0.81 (0.62, 1.06)	0.120
Proportion mediated	55.0%		20.1%	

Confounding variables: regular partner SRBS, casual partner SRBS, age 20-24, female, gay, bisexual, living with friends

*OR* Odds Ratio, *CI* Confidence Interval

^a^OR=0.89 shows that odds of STI testing in the last 12 months was 11% lower among international students compared to domestic Australian-born students (natural direct effect)

HIV knowledge accounted for nearly half (49%) of the difference in HIV testing rate between DOBS (23.4%) and DABS (22.3%) after adjusting for significant confounders (SRBS, gay, bisexual, living with friends, living alone, postgraduate). Mediation analysis of HIV testing through HIV knowledge between IS and DABS produced inconsistent results; as did mediation analysis of STI and HIV testing through STI and HIV knowledge between DOBS and IS. With a positive direct effect and negative indirect effect, a proportion mediated could not be calculated.

## Discussion

This study found that among sexually active students at five universities in South-East Queensland, Australia, regardless of student category, STI and HIV knowledge was positively associated with STI and HIV testing respectively, controlling for sexual risk behaviour and demographics. This suggests universities have an opportunity to promote increased STI and HIV testing across all student cohorts through sexual health education and health promotion programs. The authors suggest university policy makers also have a duty of care given university students are predominately from within the age range known to be disproportionately affected by STI and make a significant contribution to the Australian economy [1–3].

Exploring the relationship between STI knowledge and testing through mediation analysis revealed lower levels of STI knowledge among IS account for 55% of the lower STI testing rates, compared to DABS (after controlling for lower patterns of risk behaviour reported by IS compared to their Australian-born peers). This result suggests sexually active IS have lower levels of knowledge and its association with low testing rates may be placing this group at risk of delayed STI diagnosis. If left untreated, STI can lead to adverse short and long term health outcomes [11] and increase the risk of onward transmission to their sexual partners [28]. Lower levels of STI knowledge and STI testing were also identified in DOBS compared to DABS. Mediation analysis showed that 20% of the difference in STI testing between these two groups of domestic students was explained by STI knowledge. These are important public health finding that suggests STI testing and prevention among IS and DOBS in Australia can be improved through increasing sexual health knowledge and advocating for investment in sexual health education which considers the nuanced needs of students from CALD backgrounds.

In contrast to the differences in STI knowledge between IS compared to their domestic counterparts, we found similar average HIV knowledge across the three student groups. However, there were differences by country of origin within the IS and DOBS, for example, students from Asian regions scored lower HIV knowledge than those from other regions and their DABS peers, consistent with a previous Australian study comparing sexual health knowledge of university students born in Australia, Asia and elsewhere [6]. This suggests that while HIV knowledge was higher overall compared to STI knowledge, sexual health education should include HIV content that is targeted to the variety of backgrounds, practices and knowledge levels [29].

This study highlights the importance of sexual health education programs resulting in increased knowledge. Research suggests IS in Australia would like more sexual health education [7, 9], however more research is needed on how best to deliver this. One study noted the importance of involving CALD populations in the development and dissemination of sexual health education programs, although shame and stigma may be barriers [30]. Sexual health education should be provided through a diverse range of channels to cater for individual preferences, as well as the nuanced needs of culturally diverse priority communities. Some IS find the vast health information online overwhelming and difficult to navigate [31], while others feel more comfortable sourcing this information online [7, 9, 31]. With a large number of nationalities and cultures represented in this study, reflecting Australia’s student population and the community generally, it is important programs are tailored to cater to diverse cultural groups, languages, and learning preferences [30].

STI testing in the last 12 months among the total sample was 37%, similar to other studies of Australian university students (35%) [32] and Australian young people (36%) [33], providing confidence in the accuracy of this data. Lower rates of STI testing among IS (32%) and DOBS (34%) was not unexpected. The findings support previous research of IS and migrant populations in Australia identifying lower rates of STI testing compared to non-migrant communities [7, 14] and lower utilisation of the health services in Australia in general [34]. Of the sample, 23% had a HIV test in the last year, lower than a previous study of young people in Australia (27%) [33]. There is significant variation in both STI and HIV testing rates among sexually active students by region of birth. These findings suggest barriers to testing may vary by CALD backgrounds, which may be related to STI awareness in their home countries [6, 9].

We found IS were less likely to be sexually active and reported less risky sexual behaviour compared to DABS and DOBS. This is consistent with other studies of IS in Australia, which found IS have first sex at older ages on average and have fewer sexual partners [6, 7]. We found lower use of hormonal birth control methods and higher condom use among IS, consistent with previous research that suggests IS may use condoms as their primary contraceptive method [7, 18]. Higher risk sexual behaviour was associated with increased STI and HIV testing, consistent with research of university students [32] and young people [33] in Australia. This suggests that those at higher risk of STI are aware of it and hence are testing more. This study captured vast diversity in sexual experiences, further highlighting the need for sexual health education and testing services to avoid a ‘one size fits all’ approach; and the need to include intersectionality as an important focus. It may also be important to enhance STI and HIV-related knowledge, attitudes and practices of health professionals who support the sexual health needs of tertiary students [35].

There are some limitations of the study. Data were collected through convenience sampling and may not be representative of the university student population. However, the study had a substantial sample size and targeted efforts to recruit more male participants helped improve representativeness. The regional split of DOBS respondents is similar to population level data [4], however the IS respondents from Eastern Asia (27% vs. 40%) and Southern Asia (18% vs. 32%) are under-represented in the survey responses compared to the Australian IS population; IS respondents from outside of Asia are over-represented (35% vs. 17%) [3].

Data accuracy relies on recall of information; and this study’s focus on STI and HIV testing in the last 12 months, as opposed to lifetime testing, may have increased reliability. While the focus on recent testing better reflects experience while in Australia, this may include testing conducted offshore or before arrival for newly arrived students or students recently returned from travel home or other offshore locations. This may be magnified by visa mandated HIV testing for the small proportion of IS studying clinical disciplines [27, 36], resulting in higher IS HIV testing.

The risk of participants misreporting sensitive information was minimised by using an anonymous online survey [20, 27], but the potential for some underreporting of behaviour is still possible, so students who regard sexual health as a taboo topic may be underrepresented in the survey. Despite English literacy requirements for IS in Australia, understanding or translating sexual health terms for students from non-English speaking backgrounds may result in lost or altered meaning [37].

It is important to note that associations identified through cross-sectional surveys cannot be interpreted as causal, however, research in university students and young people suggests STI knowledge influences STI testing [16, 17]. In this analysis we have presented both a traditional regression analysis and a further analysis to assess mediating factors. We were unable to adjust for those trying to conceive (not captured); and differential item functioning of aggregate scores across student categories was not investigated.

Despite these limitations, to our knowledge, this study is the first to investigate the mediating effect of STI knowledge on STI testing in university students in Australia. It is also the first to compare these factors across the DABS, DOBS and IS cohorts.

Improving the sexual health and general wellbeing of university students, including international and overseas-born cohorts, is key to continuing and enhancing the benefits Australia gains from its diverse student population. This study showed an association between sexual health knowledge and uptake of STI and HIV testing among university students. Universities have a duty of care to provide access to sexual health information and services that meet the needs of the diverse student cohort; particularly for international students, who showed lower levels of STI knowledge and STI testing, compared to domestic students.

## Data Availability

The data for this study is available to researchers upon reasonable request, addressed to Teyl Engstrom: t.engstrom@uq.edu.au.

## References

[1] Kirby Institute (2018). HIV, viral hepatitis and sexually transmissible infections in Australia: Annual surveillance report 2018.

[2] Australian Government Department of Education Skills and Employment. Higher Education Statistics 2018 Canberra. 2019. Available from: https://www.dese.gov.au/higher-education-statistics/resources/2018-section-2-all-students.

[3] Ferguson H, Sherrel H (2019). Overseas students in australian higher education: a quick guide.

[4] Australian Government the Treasury and the Department of Home Affairs (2018). Shaping a nation: population growth and immigration over time.

[5] Simpson S, Clifford C, Ross K, Sefton N, Owen L, Blizzard L (2015). Sexual health literacy of the student population of the University of Tasmania: results of the RUSSL Study. Sex Health.

[6] Song A, Richters J, Crawford J, Kippax S (2005). HIV and sexual health knowledge and sexual experience among australian-born and overseas-born students in Sydney. J Adolesc Health.

[7] Douglass CH, Qin C, Martin F, Xiao Y, El-Hayek C, Lim MSC (2020). Comparing sexual behaviours and knowledge between domestic students and chinese international students in Australia: findings from two cross-sectional studies. Int J STD AIDS.

[8] Engstrom T, Waller M, Mullens AB, Durham J, Debattista J, Wenham K (2021). STI and HIV knowledge and testing: a comparison of domestic Australian-born, domestic overseas-born and international university students in Australia. Sex Health.

[9] Burchard A, Laurence C, Stocks N (2011). Female international students and sexual health: a qualitative study into knowledge, beliefs and attitudes. Aust Fam Physician.

[10] Rade D, Crawford G, Lobo R, Gray C, Brown G (2018). Sexual health help-seeking behavior among migrants from Sub-Saharan Africa and South East Asia living in High Income Countries: a systematic review. Int J Environ Res Public Health.

[11] Australian Government Department of Health (2018). Fourth National sexually transmissible infections strategy 2018–2022.

[12] Kong FY, Guy RJ, Hocking JS, Merritt T, Pirotta M, Heal C (2011). Australian general practitioner chlamydia testing rates among young people. Med J Aust.

[13] Coleman A, Tran A, Hort A, Burke M, Nguyen L, Boateng C (2019). Young Australians’ experiences of sexual healthcare provision by general practitioners. Aust J Gen Pract.

[14] O’Connor CC, Shaw M, Li MW, Quine S, Acculturation (2009). Sexual behaviour, risk and knowledge in Vietnamese men living in Metropolitan Sydney. Health Promot J Austr.

[15] Community of Practice for Action on HIV and Mobility (2018). HIV and mobility: priority actions.

[16] Lim MSC, Hocking JS, Aitken CK, Fairley CK, Jordan L, Lewis JA (2012). Impact of text and email messaging on the sexual health of young people: a randomised controlled trial. J Epidemiol Community Health.

[17] Denison HJ, Bromhead C, Grainger R, Dennison EM, Jutel A (2018). What influences university students to seek sexually transmitted infection testing?: a qualitative study in New Zealand. Sex Reprod Healthc.

[18] Dean J, Mitchell M, Stewart D, Debattista J (2017). Sexual health knowledge and behaviour of young sudanese Queenslanders: a cross-sectional study. Sex Health.

[19] Mitchell A, Patrick K, Heywood W, Blackman P, Pitts M (2014). 5th National Survey of Australian secondary students and sexual health 2013.

[20] Johnson AM, Copas AJ, Erens B, Mandalia S, Fenton K, Korovessis C (2001). Effect of computer-assisted self-interviews on reporting of sexual HIV risk behaviours in a general population sample: a methodological experiment. AIDS.

[21] Harris PA, Taylor R, Thielke R, Payne J, Gonzalez N, Conde JG (2009). Research electronic data capture (REDCap)—a metadata-driven methodology and workflow process for providing translational research informatics support. J Biomed Inform.

[22] Agius PA, Pitts MK, Smith AMA, Mitchell A (2010). Sexual behaviour and related knowledge among a representative sample of secondary school students between 1997 and 2008. Aust N Z J Public Health.

[23] Seal A, Minichiello V, Omodei M (1997). Young women’s sexual risk taking behaviour: re-visiting the influences of sexual self-efficacy and sexual self- esteem. Int J STD AIDS.

[24] Evans A, Sanderson M, Griffin S, Reininger B, Vincent M, Parra-Medina D (2004). An exploration of the relationship between youth assets and engagement in risky sexual behaviors. J Adolesc Health.

[25] Hernán MA (2004). A definition of causal effect for epidemiological research. J Epidemiol Community Health.

[26] Vanderweele TJ, Vansteelandt S (2010). Odds ratios for mediation analysis for a dichotomous outcome. Am J Epidemiol.

[27] Australian Government Department of Education Skills and Education. uCube – Higher Education Data Cube. Canberra. 2019. Available from: https://highereducationstatistics.education.gov.au/.

[28] Workowski KA, Bachmann LH, Chan PA, Johnston CM, Muzny CA, Park I (2021). Sexually transmitted infections treatment guidelines, 2021. MMWR
Recomm Rep.

[29] Mullens AB, Kelly J, Debattista J, Phillips TM, Gu Z, Siggins F (2018). Exploring HIV risks, testing and prevention among sub-saharan African community members in Australia. Int J Equity Health.

[30] Newton D, Keogh L, Temple-Smith M, Fairley CK, Chen M, Bayly C (2012). Key informant perceptions of youth-focussed sexual health promotion programs in Australia. Sex Health.

[31] Parker A, Harris P, Haire B (2020). International students’ views on sexual health: a qualitative study at an Australian university. Sex Health.

[32] Macphail C, Dune T, Dillon G, Rahman S, Khanam R, Jenkins L (2017). Knowledge and attitudes to sexual health and STI testing for students at an australian regional university: a cross-sectional study. J Aust New Zealand Stud Serv Assoc.

[33] Adam P, de Wit J, Ketsuwan I, Treloar C (2019). Sexual health-related knowledge, attitudes and practices of young people in Australia. Results from the 2018 Debrief Survey among heterosexual and non-heterosexual respondents.

[34] Manderson L, Kelaher M, Woelz-Stirling N, Kaplan J, Greene K (2002). Sex, contraception and contradiction among young Filipinas in Australia. Cult Health Sex.

[35] Kaladharan S, Daken K, Mullens AB, Durham J (2021). Tools to measure HIV knowledge, attitudes & practices (KAPs) in healthcare providers: a systematic review. AIDS Care.

[36] Australian Government Department of Home Affairs. Immigration and citizenship: What health examinations you need Canberra, Australia: Australian Government. 2020 Available from: https://immi.homeaffairs.gov.au/help-support/meeting-our-requirements/health/what-health-examinations-you-need.

[37] Wong HTH, Wang P, Sun Y, Newman CE, Vujcich D, Vaughan C (2023). Is sex lost in translation? Linguistic and conceptual issues in the translation of sexual and reproductive health surveys. Cult Health Sex.

